# The effect of pulse shape in theta-burst stimulation: Monophasic vs biphasic TMS

**DOI:** 10.1016/j.brs.2023.08.001

**Published:** 2023

**Authors:** Karen Wendt, Majid Memarian Sorkhabi, Charlotte J. Stagg, Melanie K. Fleming, Timothy Denison, Jacinta O'Shea

**Affiliations:** a MRC Brain Network Dynamics Unit, Nuffield Department of Clinical Neurosciences, University of Oxford, Oxford, OX1 3TH, UK; b Department of Engineering Science, University of Oxford, Oxford, OX1 3PJ, UK; c Wellcome Centre for Integrative Neuroimaging, FMRIB, Nuffield Department of Clinical Neurosciences, University of Oxford, Oxford, UK; d Wellcome Centre for Integrative Neuroimaging, Oxford Centre for Human Brain Activity (OHBA), University of Oxford Department of Psychiatry, Warneford Hospital, Warneford Lane, Oxford, UK

**Keywords:** Transcranial magnetic stimulation (TMS), Theta burst stimulation (TBS), Pulse-width modulation based TMS, TMS pulse shape, Motor plasticity

## Abstract

**Background:**

Intermittent theta-burst stimulation (i) (TBS) is a transcranial magnetic stimulation (TMS) plasticity protocol. Conventionally, TBS is applied using biphasic pulses due to hardware limitations. However, monophasic pulses are hypothesised to recruit cortical neurons more selectively than biphasic pulses, predicting stronger plasticity effects. Monophasic and biphasic TBS can be generated using a custom-made pulse-width modulation-based TMS device (pTMS).

**Objective:**

Using pTMS, we tested the hypothesis that monophasic iTBS would induce a stronger plasticity effect than biphasic, measured as induced increases in motor corticospinal excitability.

**Methods:**

In a repeated-measures design, thirty healthy volunteers participated in three separate sessions, where monophasic and biphasic iTBS was applied to the primary motor cortex (M1 condition) or the vertex (control condition). Plasticity was quantified as increases in motor corticospinal excitability after versus before iTBS, by comparing peak-to-peak amplitudes of motor evoked potentials (MEP) measured at baseline and over 60 min after iTBS.

**Results:**

Both monophasic and biphasic M1 iTBS led to significant increases in MEP amplitude. As predicted, linear mixed effects (LME) models showed that the iTBS condition had a significant effect on the MEP amplitude (χ^2^ (1) = 27.615, p < 0.001) with monophasic iTBS leading to significantly stronger plasticity than biphasic iTBS (t (693) = 2.311, p = 0.021). Control vertex iTBS had no effect.

**Conclusions:**

In this study, monophasic iTBS induced a stronger motor corticospinal excitability increase than biphasic within participants. This greater physiological effect suggests that monophasic iTBS may also have potential for greater functional impact, of interest for future fundamental and clinical applications of TBS.

## Highlights

• Plasticity induction via pulse-width modulation-based TMS.
• Both mono- and biphasic theta burst stimulation induced motor plasticity.
• Monophasic intermittent TBS induced a larger plasticity effect than biphasic.

## 1 Introduction

Transcranial magnetic stimulation (TMS) is a non-invasive tool for neuroscientific research and is increasingly used for diagnosis and therapy in clinical practice [1]. It uses the fundamental principles of magnetic induction to modulate the nervous system: a brief electric current is applied to a stimulating coil, which creates a rapidly changing magnetic field that induces a voltage in the brain tissue underneath the coil. When applied repeatedly, TMS can induce plasticity – causing a change in cortical excitability of the targeted brain area that outlasts the stimulation period [2].

Different stimulation waveforms have been shown to recruit different neural populations, have different excitation thresholds, and have different effects on corticospinal excitability [[3], [4], [5], [6], [7], [8]]. However, the range of stimulation pulses and patterns that can be generated by conventional TMS devices is limited by the device hardware and is usually confined to either monophasic or biphasic damped cosine pulses, where the exact shape and length of the pulse is determined by the resonance between the device components [9]. For monophasic pulses, the current flow is commonly dampened half-way through the cycle of the cosine pulse by letting the current flow through a shunting diode and dissipating the energy through a resistor. This restricts not only the choice of TMS pulse waveforms and widths but also the achievable repetition rates [10]. For example, one class of repetitive TMS protocols, widely used for plasticity induction in fundamental research and clinical applications, that is constrained by these hardware limitations, is theta burst stimulation (TBS). During TBS, bursts of 3 pulses are applied at 50 Hz and repeated every 200 ms [11]. In intermittent (i)TBS, a largely excitatory protocol, these triplets are applied for 2 s followed by an 8 s break and then repeated again, for 600 pulses in total [11]. To sustain these repetition rates, large amounts of energy need to be recovered after each stimulation pulse, and so TBS can usually only be delivered via a conventional TMS device using biphasic stimulation pulses.

Monophasic pulses are thought to more selectively recruit cortical neurons and have been shown to more strongly modulate cortical excitability than biphasic pulses when used in other repetitive TMS protocols [1,6,8,12,13]. For example, in quadripulse stimulation (QPS), bursts of four pulses are applied at inter-stimulus intervals of 1.5–1250 ms, repeated every 5s over 30 min [14]. A study comparing the after-effects of monophasic and biphasic QPS found that monophasic QPS induced stronger and longer lasting after-effects compared with biphasic QPS [13]. Such findings lead to the hypothesis that applying TBS with monophasic pulses may be more effective than existing biphasic TBS.

Recent technological developments of TMS devices using switching circuits, rather than the conventional resonance circuits, have allowed more control over TMS parameters and better energy recovery from the stimulation pulses [9,[15], [16], [17]]. The programmable (p)TMS, a TMS device developed within our research group, which uses pulse-width modulation (PWM) to control cascaded inverters, enables more control over the pulse shapes by approximating a reference pulse of arbitrary shape using discrete voltage levels [9]. Previous evidence from computational modelling and an in-human physiology study indicated that the approximations of conventional pulse shapes generated using the pTMS have similar effects on the motor corticospinal excitability of healthy volunteers as the pulses generated by a conventional TMS device [18,19]. Additionally, the pTMS device recovers energy effectively after each pulse, making the generation of monophasic TBS possible.

In this study, we use the pTMS device to generate monophasic and biphasic iTBS and compared the effects on motor corticospinal excitability of healthy volunteers. We predicted that monophasic iTBS would produce a larger plasticity effect (higher MEP amplitudes) than biphasic iTBS. To control for intra- and inter-individual variability, we also applied the same stimulation to the vertex in a control condition.

## 2 Materials and methods

### 2.1 Ethical approval

This study and the use of the pTMS device in this study were approved by the local ethics committee at the University of Oxford (Central University Research Ethics Committee, R75180/RE008). All participants gave their written informed consent prior to participating and were compensated for their time with £10/hr.

### 2.2 Participants

30 healthy volunteers (16 females, aged 19–33 years, mean age 24.5 years) participated in one familiarisation session followed by three data collection sessions for this single-blind, within-participants crossover study. All participants were right-handed as assessed by the Edinburgh Handedness Inventory [20]. Participants were screened to rule out any current significant medical condition and any contraindication to TMS in line with international safety guidelines [21].

### 2.3 Transcranial magnetic stimulation

The iTBS intervention protocols were applied using the pTMS stimulator. The pulse waveforms generated by the pTMS stimulator were designed to closely approximate the conventional biphasic and monophasic pulses generated by a Magstim Rapid^2^ and a Magstim 200, respectively (see Ref. [18] for a detailed comparison). To measure the motor corticospinal excitability before and after iTBS, a Magstim 200 stimulator (Magstim Co., UK) was used to generate monophasic single-pulse TMS to induce MEPs. A 70 mm figure-of-8 coil (Magstim Co., P/N 9925–00) was used to deliver all stimulation. Owing to coil overheating, for participants with resting motor thresholds (RMTs) above 43% of the maximum stimulator output (MSO) of the Magstim 200 (N = 3), one coil was used for MEP measurement and a second coil for iTBS. For all other participants, the same coil was used throughout.

Prior to the three test sessions, there was an initial familiarisation session for participants naïve to TMS, where TMS was introduced to the participant and the hotspot and thresholds for the different parameters and devices were found. The pTMS stimulator's maximum pulse amplitude is 1600 V, compared to the maximum amplitude of the Magstim Rapid^2^ and Magstim 200, which are approximately 1650 and 2800 V, respectively [19]. Therefore, to ensure the pTMS stimulator could generate iTBS at 70% of the RMT for both monophasic and biphasic pulses, individuals with RMTs above 47% MSO of the Magstim 200 were excluded from any further participation in the study (N = 3).

**Fig. 1:**
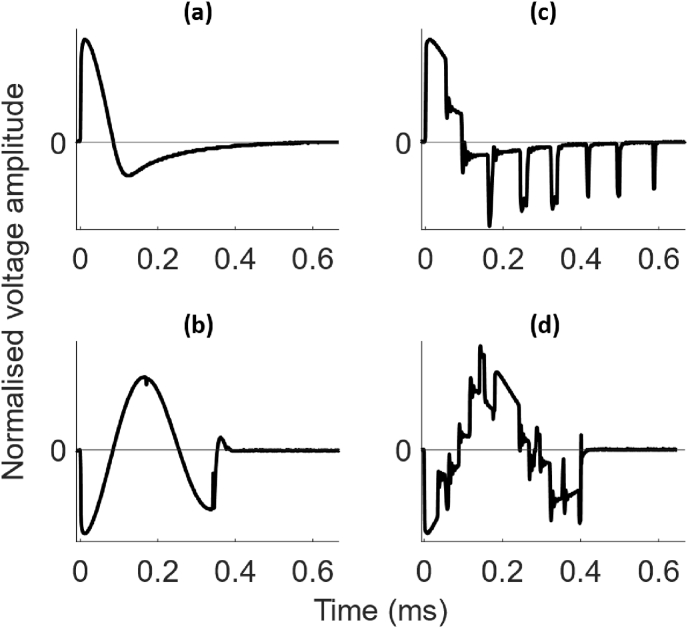
Recordings of the Magstim and pTMS pulse waveforms. Normalised voltage waveforms of **(a)** the Magstim 200 and **(b)** the Magstim Rapid^2^ stimulators which were used as the reference pulses to generate **(c)** the monophasic pulses and **(d)** the biphasic pulses with the pTMS stimulator. The direction of the biphasic pulses in (b) and (d) was adjusted such that the dominant second phase of the pulse matched the direction of the monophasic pulse. All recordings were measured using a pick-up coil at a sampling rate of 1 MS/s.

During the familiarisation and test sessions, participants were seated in a chair with their arms resting on a pillow on top of a table in front of them. The ‘motor hotspot’ of the left primary motor cortex was defined as the scalp location over which the lowest TMS pulse intensity elicited MEPs in the relaxed first dorsal interosseous (FDI) muscle of the right hand. For all TMS pulses, the coil was held by the operator and oriented at 45° to the midline with the handle pointing posteriorly, which results in a posterior-anterior current flow in the brain for the monophasic pulse. The direction of the biphasic pulse was reversed via the software, such that the direction of the dominant second phase of the pulse matched the current flow of the monophasic pulse (Fig. 1) [3,5]. A Brainsight neuronavigation system (Rogue Research Inc., Montreal, Canada) was used to record the motor hotspot and for continuous tracking to maintain the position and orientation of the coil. Surface electromyography (EMG) of the right FDI was recorded in a belly-tendon montage (see supplementary information (SI) page 1 for details).

The RMT, defined as the minimum intensity required to evoke an MEP of ≥50 μV peak-to-peak amplitude in 5 out of 10 consecutive trials, was determined by applying 10 pulses at each intensity and inspecting the EMG traces visually in real time for each device and pulse shape. To find the RMT, the pulses were triggered automatically via scripts in Signal version 7.01 (Magstim device) and Control desk (pTMS device) software at inter-pulse intervals of 5 s (±15%).

Baseline excitability before and after iTBS was quantified by blocks of 30 single TMS pulses at 120% of the RMT at inter-pulse intervals of 5 s (±15%). The iTBS protocol consisted of 600 either monophasic or biphasic pulses applied at 70% of the RMT [22].

### 2.4 Procedure

**Fig. 2:**
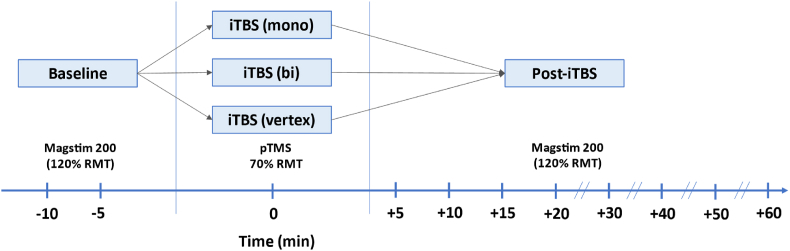
Overview of study design representing the flow of data collection at each visit. Each participant received each iTBS condition on separate days (at least one week apart) in counter balanced order. Baseline MEPs in response to single-pulse TMS (30 pulses applied at 120% of the resting motor threshold) were collected in 2 separate blocks 10 and 5 min before the start of the iTBS administration. iTBS was applied to the primary motor cortex (M1 condition) or the vertex (control condition) using monophasic or biphasic pulses for 190s. After iTBS, MEPs were collected every 5 min for the first 20 min and then at 10-min intervals up to 60 min post-iTBS.

The familiarisation and data collection sessions were at least one week apart, with each participant's total duration of participation not exceeding 10 h. During each data collection session, the timeline was as follows (Fig. 2). After confirmation of the hotspot and the motor threshold, two baseline blocks of MEPs were recorded 5 min apart (30 pulses per block). iTBS was applied 10 min after the start of the first baseline block and follow-up blocks were recorded every 5–10 min after the start of the iTBS protocol over the following hour (at 5, 10, 15, 20, 30, 40, 50 and 60 min). For each participant, two sessions were M1 conditions, where iTBS was applied to the motor hotspot, and one session was a control condition, where iTBS was applied to the vertex (similar to Ref. [23]). In the control condition, participants were randomized to receive either monophasic iTBS (N = 14) or biphasic iTBS (N = 16). The coil was lifted from the participants' heads between each stimulation block and participants were instructed to keep their hands as relaxed as possible throughout.

The three test sessions were performed at the same time of day for each participant and the session order was randomised and counterbalanced. The participants were blinded to the stimulation condition.

### 2.5 Data processing

The data were processed in Python using custom scripts. Since muscle activation can influence MEP amplitude, trials were excluded if the root mean square (RMS) of the EMG trace in the 90 ms before the TMS stimulus (excluding the 5 ms preceding the pulse to avoid contamination from the TMS artefact) exceeded 0.02 mV. The EMG recordings included burst noise, a type of electrical noise characterised by sudden transitions between discrete voltage levels, with a peak-to-peak amplitude of 0.039 mV. To distinguish between this noise and any muscle activation, the pre-stimulus RMS was compared to the RMS during the silent period after the MEP (60–90 ms after the TMS pulse), where no muscle activity is expected but electrical noise may be present. Any trials where the pre-stimulus RMS was 0.005 mV larger than the RMS of the silent period or where the MEP was indistinguishable from electrical noise (i.e. smaller than a peak-to-peak amplitude of 0.039 mV) were also excluded. The peak-to-peak MEP amplitude was calculated in the 15–60 ms time window after the TMS pulse was applied. At the individual participant level, we first tested whether MEP amplitudes within each block were normally distributed. Across participants, over 55% of the stimulation blocks were not (Shapiro-Wilk <0.05). This was not improved by log-transformation of the data. Hence, the median (rather than the mean) MEP amplitude was calculated for each time point block per participant, as the median is a more appropriate measure of central tendency of a non-normal distribution. In addition, it also helped reduce the effect of outliers without requiring outlier removal. Supplementary analyses using the mean MEP amplitude for each time point block per participant can be found in the supplementary information (page 6), for comparison. On the group level, the data were not normally distributed (100% of the stimulation blocks). Log transformation helped render the MEP distribution more normal (only 0.03% of the stimulation blocks not normally distributed). Therefore, all MEP data were log transformed (base 10). The log-transformed medians from the two baseline blocks were averaged for each participant to give one baseline score per session. Grand-average log-transformed MEP data were calculated for each condition by averaging over all post-iTBS blocks (5–60 min).

### 2.6 Data analysis

All analyses were performed on the log-transformed absolute MEP amplitudes, rather than on data transformed into post-iTBS ratio changes from baseline. Absolute values are preferable to ratios when analysing change, as ratios have been shown to misrepresent physiological processes and to lead to false inferences about group differences [24]. Since participants were randomized to receive either mono- or biphasic iTBS in the control vertex condition, analyses tested for any difference between the two. As there was no difference, the data were combined into a single control vertex condition for analysis (see SI, page 7). To test for significant differences in pre-iTBS baseline excitability, repeated measures analysis of variance (rmANOVA) with factors Time (baseline blocks 1 and 2) and Condition (monophasic, biphasic, control) was used to compare the peak-to-peak MEP amplitudes of the two baseline blocks within and across conditions. Resting motor thresholds obtained using the Magstim 200 were also compared across testing sessions using rmANOVA with the factor Condition (monophasic, biphasic, control).

To test whether iTBS induced plasticity (i.e. a predicted overall increase in MEP amplitude post-iTBS relative to pre-iTBS), the block-wise log-transformed data of each participant were averaged over all post-iTBS time points to yield a single mean post-iTBS MEP score to contrast with each individual's baseline score pre-iTBS. Grubb's test on these data did not reveal any outliers at the group level. These grand-average MEP data were first compared within-condition against the baseline and next across conditions using rmANOVA with factors Condition (monophasic, biphasic, control) and Time (baseline, post-iTBS average). To compare relative plasticity induction across conditions, post hoc comparisons using pairwise t-tests were conducted. Holm-Bonferroni correction for multiple comparisons was applied. Greenhouse–Geisser correction was applied where appropriate and effect sizes are reported using Cohen's dz for pairwise t-tests [25]. To test whether differences between conditions were outlier driven, a supplementary responder analysis of the grand average of the monophasic and biphasic M1 conditions was also conducted, where responders were defined as having a grand average change from baseline (post-iTBS grand average – baseline) above 0 and non-responders below 0, similar to the responder analysis in Ref. [26].

To make use of the full MEP time-course data, complementary analyses were also run using linear mixed effects (LME) models. One advantage of this approach over rmANOVA is that it enables the inter- and intra-participant variability in the baseline data to be modelled in the analysis, as opposed to accounting for the baseline variability by calculating MEP percentage change scores, an approach which often fails to correctly model physiological processes [24]. In contrast to the previous grand-average MEP analysis, the MEP amplitudes at each post-iTBS time point were used, without grand averaging over the time points. In the LME models, Baseline MEP amplitude, Time (5–60 min post-iTBS) and iTBS condition (monophasic M1, biphasic M1, vertex) were modelled as fixed effects while participants were modelled as a random effect. This allowed model intercepts to differ for different participants. To test for an effect of iTBS condition, likelihood ratio testing was used to contrast two models – one that included iTBS condition as a factor in the model versus a model without the iTBS condition. The χ^2^ statistics representing the difference in deviance between the two models are reported, together with the p values calculated by the *anova* function using the Satterthwaite's method for denominator degrees-of-freedom and F-statistic [27]. Post hoc comparisons across conditions were used to test for differences between the monophasic and biphasic M1 conditions and the vertex condition. All linear mixed effects models were created and analysed using purpose-written R code using the LME4 and lmerTest packages [27,28]. The post hoc comparisons were conducted using the emmeans package in R [29] using Holm-Bonferroni correction for multiple comparisons. The significance level was set to 0.05 for all analyses.

## 3 Results

### 3.1 No differences in RMTs or MEP amplitude at baseline

Resting motor threshold intensities did not differ between conditions (F (2, 58) = 0.43; p = 0.65). Also, a two-way rmANOVA showed that within sessions there were no significant differences between the first and second baseline measurements (F (1, 29) = 0.004; p = 0.950), nor did these differ across the iTBS sessions (F (2, 58) = 1.774; p = 0.184; Supplementary Fig. S1). Thus, these analyses confirmed that participants were tested at comparable levels of motor corticospinal excitability prior to iTBS in all three conditions.

### 3.2 Both active iTBS conditions led to increased motor corticospinal excitability

**Fig. 3:**
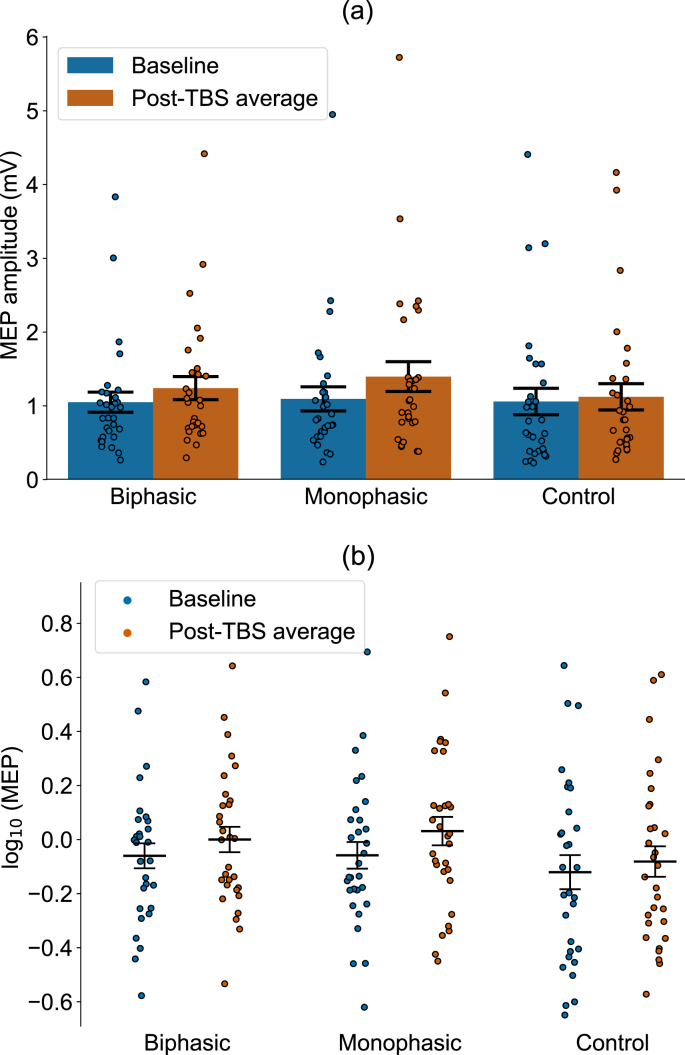
Group mean grand-average MEP amplitude compared to baseline, averaged across the 60-min post-iTBS time period for the M1 (monophasic and biphasic) and the control (vertex) conditions. **(a)** Absolute MEP values are shown in mV for ease of interpretation. As the data were not normally distributed, all analyses were performed on log-transformed data (which resolved the skew). Log-transformed data are visualized in **(b)**. Individual participants are indicated by dots, the bars indicate group means and the error bars represent ±1 standard error of the mean. For more detailed visualization of individual responses across the different iTBS conditions, the reader is referred to Figs. S2 and S3.

Fig. 3a shows the baseline and group mean average MEP amplitude over the follow-up period (5–60 min post-iTBS) for each condition for the raw MEP data (in mV). Since the data were not normally distributed (note the positive skew), they were log transformed, which resolved this problem. Fig. 3b shows the log-transformed data that were entered into the analysis. RmANOVA revealed a significant effect of Time (F (1, 29) = 23.738; p < 0.001) and Condition (F (2, 58) = 4.389; p = 0.023) but no interaction of Time and Condition (F (2, 58) = 1.686; p = 0.200). To investigate the effect of time within each condition, Holm-Bonferroni corrected pairwise comparisons of baseline and post-iTBS averages were conducted for each condition. As predicted, biphasic iTBS induced a significant increase in MEP amplitude (t (29) = 4.125, p < 0.001; ΔM: 0.19 mV, SEM: ±0.05 mV; dz = 0.753), confirming a plasticity effect. Monophasic iTBS also induced significant plasticity (t (29) = 4.236, p < 0.001; ΔM: 0.30 mV, SEM: ±0.07 mV; dz = 0.773). In the control condition, iTBS over the vertex did not lead to a significant MEP increase (t (29) = 1.604, p = 0.12; ΔM: 0.06 mV, SEM: ±0.05 mV; dz = 0.293). To contrast across conditions, Holm-Bonferroni corrected pairwise comparisons showed that only the monophasic plasticity effect was significantly larger than the control condition (t (29) = 2.767; p = 0.029; dz = 0.505; monophasic vs biphasic: t (29) = 0.677; p = 0.504; dz = 0.124; biphasic vs control: t (29) = 1.927; p = 0.128; dz = 0.352). The number of responders in both M1 conditions was 26, the number of non-responders was 4. As shown in Fig. S4, the non-responders in the monophasic M1 condition did not correspond to the non-responders in the biphasic M1 condition and 73% of participants showed consistent responses (i.e. the predicted MEP increase) between the two conditions. In summary, this analysis indicates that both monophasic and biphasic M1 iTBS induced plasticity, with only the monophasic iTBS leading to larger plasticity induction than the control condition when averaging over post-iTBS time points. The effect was not outlier driven.

### 3.3 TMS pulse shape affects the iTBS plasticity effect

**Fig. 4:**
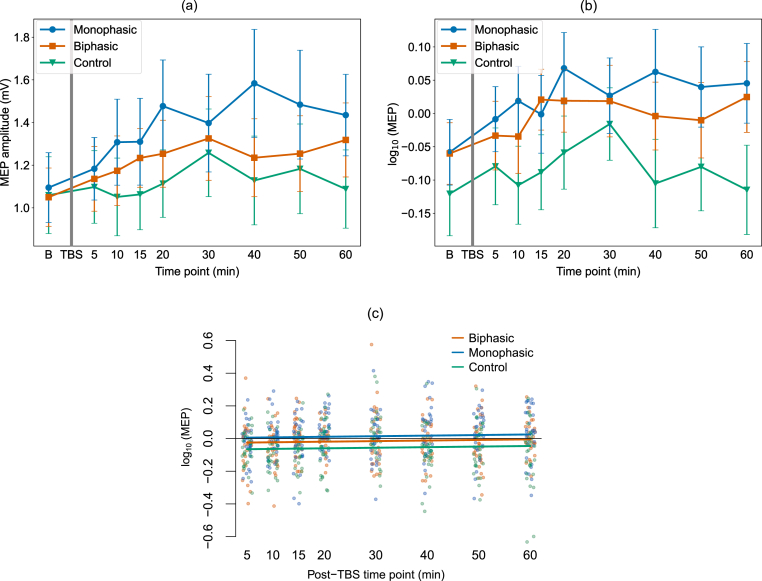
Group mean motor evoked potential amplitude over time for the different iTBS conditions. **(a)** The mean MEP amplitude for the monophasic and biphasic M1 iTBS conditions and the control condition are shown for the baseline and across the post-iTBS time points. Absolute MEP values are shown in mV for ease of interpretation. As the data were not normally distributed, all data were log-transformed (which resolved this problem). Log-transformed data were entered into analysis, which is visualized in **(b)**. Error bars indicate standard error of the mean. **(c)** Visualization of the fit of the linear mixed effect model (including the fixed effect of iTBS condition) to the data from the monophasic and biphasic M1 iTBS conditions and the vertex condition. The model was fit to the log-transformed absolute MEP amplitudes. The baseline data (not shown here) were modelled as a separate fixed effect. Solid lines show the model predictions, single dots show partial residuals as generated using the ‘visreg’ function in R. Supplementary Fig. S5 shows the three iTBS conditions each on individual plots for closer inspection.

Fig. 4a shows the full time-course MEP amplitudes across the 60-min follow-up period for each stimulation condition using the raw data. As these were not normally distributed, they were log-transformed, which resolved the positive skew. Fig. 4b shows the log-transformed data which were used in the analysis as such data were normally distributed. Individual plots for each participant, timepoint and condition are shown in Supplementary Fig. S2. To take the full time-course data into account for the analysis, the LME models with and without the fixed effect of iTBS condition were compared using likelihood ratio testing, which showed that the iTBS condition (monophasic M1, biphasic M1, vertex) had a significant effect on the MEP amplitude (χ^2^ (1) = 27.615, p < 0.001). Fig. 4c shows the model fit of the LME model including the fixed effect of iTBS condition for the different iTBS conditions. Post-hoc comparisons revealed significant differences between the monophasic and biphasic M1 conditions (t (693) = 2.311, p = 0.021), as well as the M1 conditions and the vertex condition (biphasic vs vertex: t (704) = 3.077, p = 0.004, monophasic vs vertex: t (704) = 5.345, p < 0.001). This analysis confirms that when considering the full MEP time-course data, monophasic iTBS induced a stronger motor corticospinal excitability increase than biphasic iTBS, and both active conditions induced stronger increases than the vertex control condition.

## 4 Discussion

In this study, both monophasic and biphasic iTBS applied to the primary motor cortex increased motor corticospinal excitability as measured using MEPs. The application of iTBS using the pulse-width modulation based TMS device resulted in excitability increases, as expected from studies applying iTBS using conventional resonance-based devices. On the group level, monophasic iTBS induced larger plasticity effects than conventional biphasic iTBS, confirming the importance of pulse shape in repetitive TMS protocols. iTBS applied to the vertex in the control condition did not lead to a change in MEPs. A responder analysis showed that for the majority of data, participants responded in both conditions but overall, more strongly in the monophasic condition.

### 4.1 Influence of high-frequency components in the stimulation pulses

In this study, iTBS was applied using PWM which includes more high-frequency components in the stimulation pulse than conventional pulses. While previous studies showed similar effects of PWM pulses compared to conventional single pulses [18,19], few studies have investigated the effects of PWM on plasticity induction via repetitive TMS. A study comparing the effects of 1 Hz rTMS on cortical excitability using rectangular pulses applied using the cTMS device and conventional biphasic pulses found stronger effects with rectangular bidirectional and unidirectional pulses than with the conventional biphasic pulses [30]. The pulses applied using the pTMS device in this study approximate the conventional monophasic and biphasic pulses more closely than rectangular pulses do, however the additional high-frequency components in the pTMS pulses may also contribute to the effects observed in this study. In particular, the current study did not track a return to baseline of the MEP amplitude at the end of the sampling interval at 60 min in the active iTBS conditions. For comparison, some previous studies applying conventional biphasic iTBS reported elevated MEP amplitudes up to 50 min after iTBS (e.g. Refs. [31,32]). A longer post-iTBS sampling window would be required in future studies to estimate how long the plasticity effect lasts with monophasic and PWM iTBS.

### 4.2 Choice of probing pulses

To measure the changes in motor corticospinal excitability, single pulses were applied at 120% of the RMT, similar to previous work such as [33,34]. Other studies, including the early iTBS studies [35], did not use % of RMT as a baseline/probe, but instead used the individualized intensity of TMS needed to reliably elicit MEPs of 1 mV on each trial. However, this approach can suffer from floor or ceiling effects across individuals (e.g. in some participants, TMS may not induce peak-to-peak amplitudes much higher than 1 mV) which can contribute to the high variability across participants [36]. Therefore, plasticity effects have been suggested to best be probed at a percentage of the resting motor threshold to take into account this difference in the input-output characteristics of the participants [37]. This reduces the risk of ceiling and flooring effects on plasticity but entails higher variability between participants at baseline. This was accounted for in the analysis of this study by including the baseline as a factor in the LME model.

Consistent with the literature, monophasic single pulses applied using a conventional Magstim 200 were used to measure MEPs pre/post-iTBS. While this allowed the direct comparison of effects across conditions, monophasic and biphasic pulses may activate different neural populations during TBS. Using monophasic pulses therefore limits the ability to probe the potentially different neural populations activated by biphasic TBS [12,38]. However, studies using both monophasic and biphasic pulses to assess plasticity effects found the results of using both pulse shapes highly comparable [39,40].

### 4.3 Directionality of pulse currents

The direction of the current induced in the brain affects which neuron populations are activated and influences the size of the motor threshold [3,5]. Early studies using epidural recordings in human participants have shown that anterior-posterior and posterior-anterior monophasic stimulation likely activate different sites or different neuron populations, in particular at low intensities [41]. Biphasic pulses with posterior-anterior current flow in their second phase have been shown to recruit descending volleys, similar to posterior-anterior monophasic pulses, however cortical activation patterns depend on relative thresholds and intensities of the different pulse phases [42]. In this study, the monophasic pulses were applied to induce currents in the posterior-anterior direction both for single pulses and for the iTBS protocol, as this has been shown to have the lowest thresholds [5] and may therefore be the best current direction to use when the aim is to increase motor corticospinal excitability, as here. The biphasic pulses were applied to match this current direction in the second phase of the pulse, as this is thought to be the dominant activating phase of the biphasic pulse [5]. A previous study showed that the current direction of biphasic pulses had a significant effect on corticospinal excitability when using continuous (c) but not intermittent TBS [43]. However, another study found no difference in effects between different current directions in cTBS [44]. Another variable of potential interest is the current direction used in the probing pulses, although a previous study found no effect of current direction on MEP measures of cTBS response when using 1 mV probing pulses [40]. Future studies should explore the effects of different current directions of the different pulse shapes, and different intensities, to determine the optimal current direction for plasticity induction.

### 4.4 Further considerations to improve TBS

Monophasic pulses approximating the pulse shape of conventional stimulators were used in this study, but other pulse shapes or widths may cause larger effects in TBS and other repetitive TMS protocols. With the newer TMS devices such as the pTMS device used in this study, researchers gain the ability to investigate more parameters to optimise the effects of TBS. Other studies looking at the number of pulses per burst [45], different stimulation intervals [38,46] as well as the total number of pulses [47,48] show further possible avenues to increase the plasticity effects induced by the stimulation.

### 4.5 Vertex stimulation as control condition

In the control condition, iTBS was applied to the vertex, using the same parameters as in the M1 conditions. We chose to apply real iTBS to the vertex to achieve an active control condition in which the same stimulation is applied (but at an anatomical control site) and similar skin sensation and audio effects are experienced [23,49]. The purpose of the vertex condition was to establish anatomical specificity (of M1 iTBS effects) and to quantify intrinsic intra- and inter-participant variability in MEP amplitude fluctuations over the same measurement time period as in the M1 conditions. While there were fluctuations in MEP amplitude over time in the post-iTBS blocks in the control condition, none of these were significant. At the individual level, such MEP fluctuations are likely due to non-specific psychological factors, such as attention and fatigue. In addition, as the brain was actively stimulated in the vertex condition, albeit in a different location, brain network effects may have also influenced the MEP results. However, the active control condition showed no systematic change post-iTBS, reflected in non-significant analyses, indicating that any apparent visual changes (Fig. 4a) reflect weak and variable non-specific MEP fluctuations over time.

### 4.6 Limitations

Due to the technical setup of the study, it was conducted in a single-blinded manner, where the participants were blinded to the condition and the study hypothesis, but the experimenters were aware of the stimulation condition. This was partially due to the online programming needed for the custom-made pTMS device to generate monophasic or biphasic pulses and the fact that the coil was placed in a different location during the control condition. The pTMS stimulator's limit on its maximum pulse amplitude necessitated the exclusion of some participants with high thresholds. This is a limitation of the current prototype. However, the maximum output intensity of future generations of the device can be increased by cascading additional H-bridges. The interval between probing pulses in this study was 5 s (±15%) which may lead to confounds due to anticipation or carry-over effects. However, as the same intervals were used in all conditions, these confounds are unlikely to account for differences between conditions. Additionally, the test sessions were 2–3 h long, during which experimenters interacted with the participants, albeit as little as possible, which may have had an influence on the results and the MEP variability, though the use of a within-participants crossover design should help to mitigate this potential issue to some extent.

## 5 Conclusions

This study confirms that the pulse shape affects the group-level plasticity effects induced after iTBS, with monophasic pulses leading to larger increases in MEP amplitude than conventional biphasic. This adds to the literature exploring improvements of the TBS protocol in the hope of enhancing plasticity induction. As technology advances and the limitations of current systems are addressed, these findings hold promise for applications in basic neuroscience and medical practice such as depression therapy.

## Rights retention

For the purpose of Open Access, the authors have applied a CC BY public copyright licence to any Author Accepted Manuscript version arising from this submission.

## Data availability statement

The data and relevant scripts are available on the Open Science Framework (https://osf.io/9e62f/).

## CRediT authorship contribution statement

**Karen Wendt:** Conceptualizsation, Methodology, Investigation, Formal analysis, Writing – original draft. **Majid Memarian Sorkhabi:** Methodology, Investigation, Resources, designed and built pTMS device, Writing – review & editing. **Charlotte J. Stagg:** Methodology, Writing – review & editing. **Melanie K. Fleming:** Methodology, Writing – review & editing. **Timothy Denison:** Conceptualizsation, Resources, designed and built pTMS device, Writing – review & editing, Supervision, Funding acquisition. **Jacinta O'Shea:** Conceptualizsation, Methodology, Writing – review & editing, Supervision, Funding acquisition.

## Declaration of competing interest

The authors declare the following financial interests/personal relationships which may be considered as potential competing interests: Timothy Denison, Majid Memarian Sorkhabi and Karen Wendt have pending patent applications for TMS device circuits and control algorithms. Timothy Denison and Karen Wendt have received grant funding (including an 10.13039/501100000265MRC iCASE studentship) and materials through collaboration agreements from Magstim Ltd. Majid Memarian Sorkhabi is currently employed by Magstim Ltd. Jacinta O'Shea has acted as a consultant for Welcony Inc. And serves on the Scientific Advisory Board of Plato Science. Charlotte J. Stagg and Melanie K. Fleming have no conflicts of interest.
