# Supplementary material for: The effect of pulse shape in theta-burst stimulation: Monophasic vs biphasic TMS
Source: Brain Stimul. 2023 Jul-Aug;16(4):1178–85. doi: 10.1016/j.brs.2023.08.001 (PMC10444700; doi:10.1016/j.brs.2023.08.001)
Supplement: Multimedia component 1 [file mmc1.docx]

**Supplementary File for “*The effect of pulse shape in theta-burst stimulation: monophasic vs biphasic TMS*”**

Karen Wendt, Majid Memarian Sorkhabi, Charlotte J. Stagg, Melanie K. Fleming, Timothy Denison, Jacinta O’Shea

**Electromyography setup**

Surface electromyography (EMG) of the right FDI was recorded using disposable neonatal ECG electrodes (Kendall, Cardinal Health, UK) in a belly-tendon montage with a ground electrode over the ulnar styloid process. EMG signals were sampled at 10 kHz, amplified with a gain of 1000, filtered (10 Hz - 1000 Hz) and recorded using a D440 Isolated Amplifier (Digitimer, Welwyn Garden City, UK), a Micro1401 (Cambridge Electronic Design, Cambridge, UK) and Signal software version 7.01 (Cambridge Electronic Design). The unwanted 50 Hz line noise was attenuated by subtracting a replica of the measured noise from the input signal using a HumBug Noise Eliminator (Digitimer).

**Supplementary figures**

**
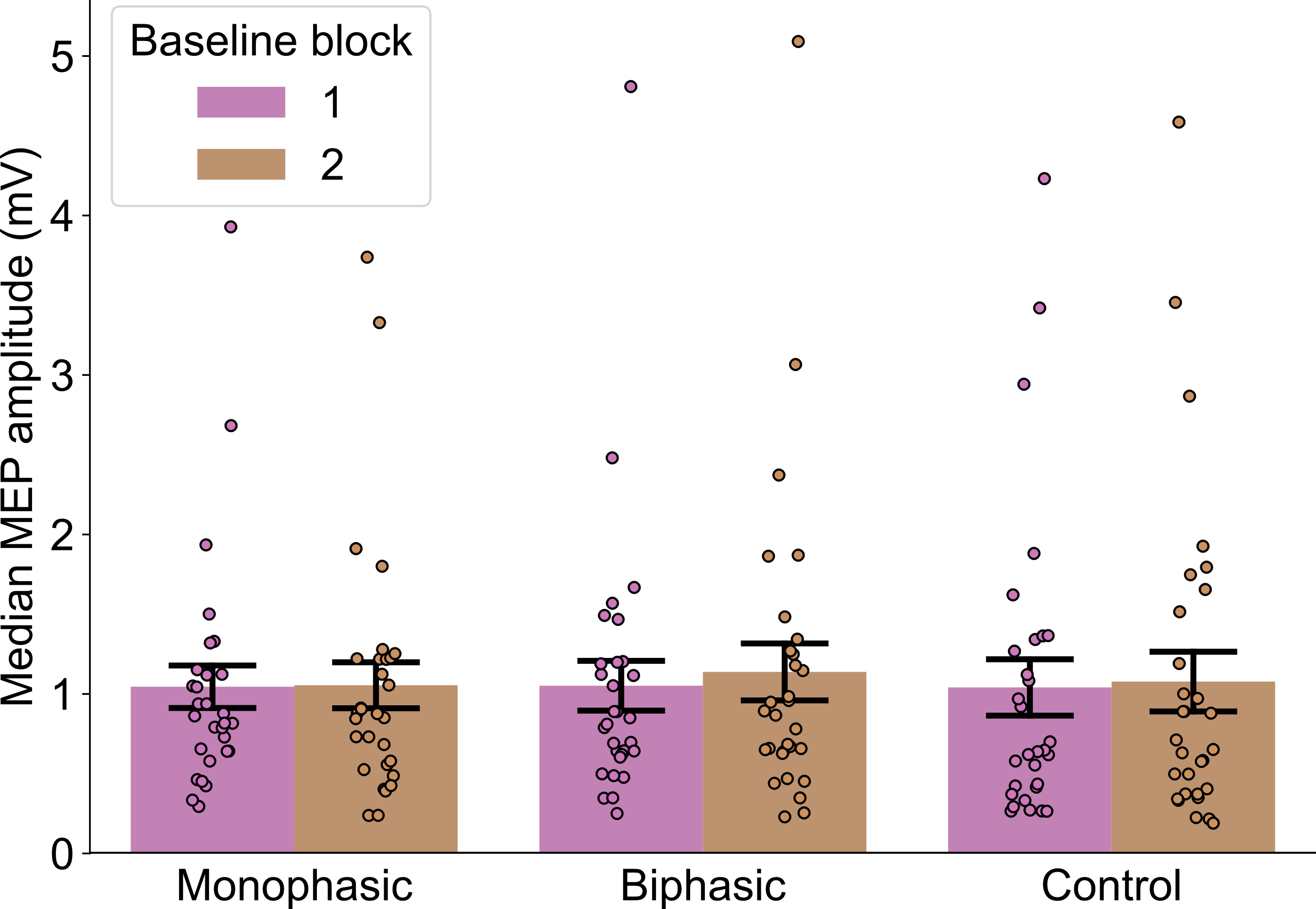
**

*Fig. S1: Group mean of the median peak-to-peak MEP amplitude for each of the two baseline blocks averaged across participants in each condition. Each baseline block consisted of MEPs elicited by 30 single TMS pulses at 120% of the resting motor threshold. The group mean MEP amplitude was close to 1 mV for each condition and block, but the median amplitudes of individual participants varied. Individual participants medians are indicated by dots, the bars indicate group means and the error bars represent ± 1 standard error of the mean.*

*
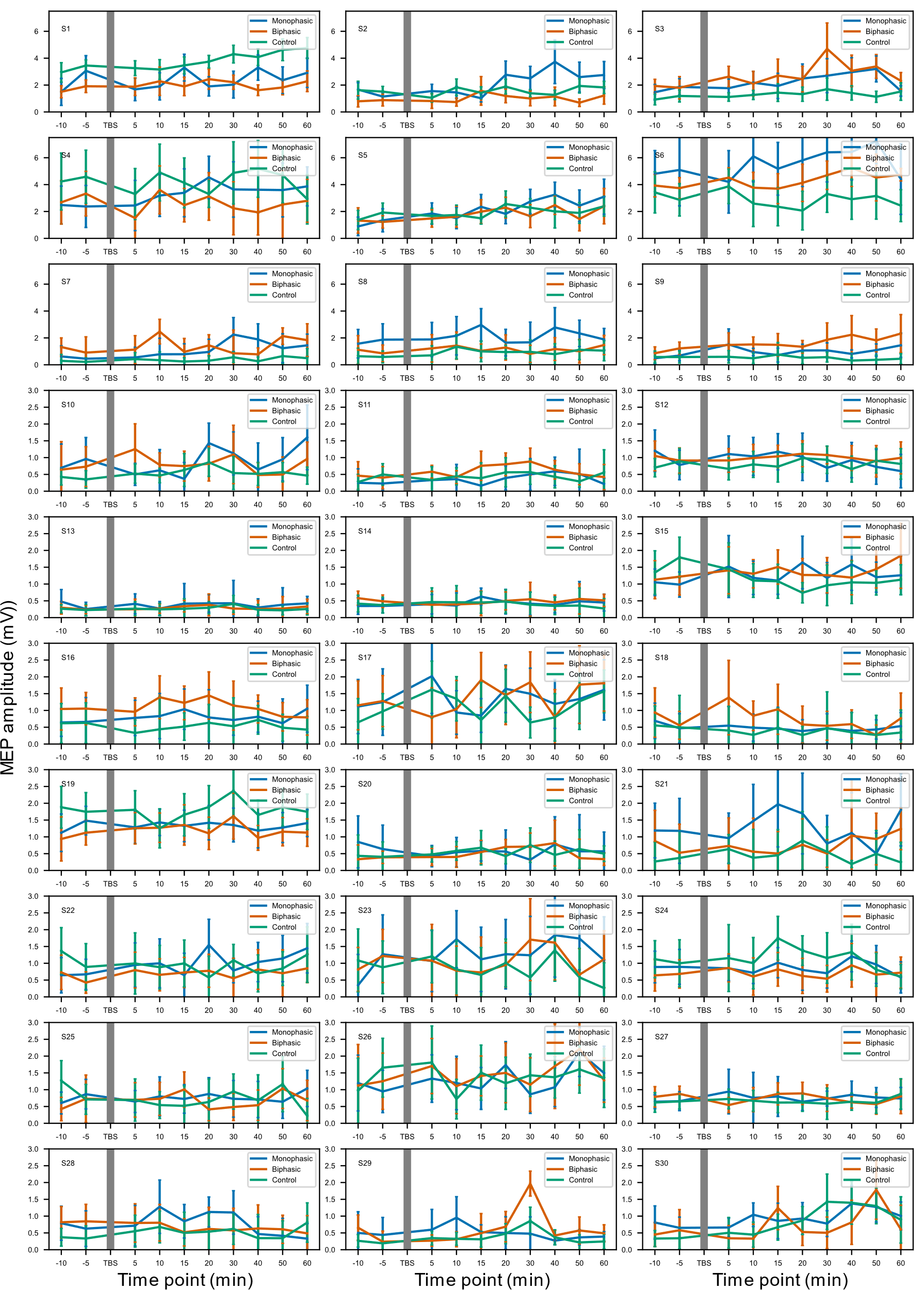
*

*Fig. S2: Median block-wise MEP amplitudes for all participants and TMS conditions. Each plot shows the data of one participant across all data collection time points in all iTBS conditions in mV. The error bars represent the standard deviation. Note: the y-axis range has the same scale for S1-S9, and a different range for S10-S30.*

**
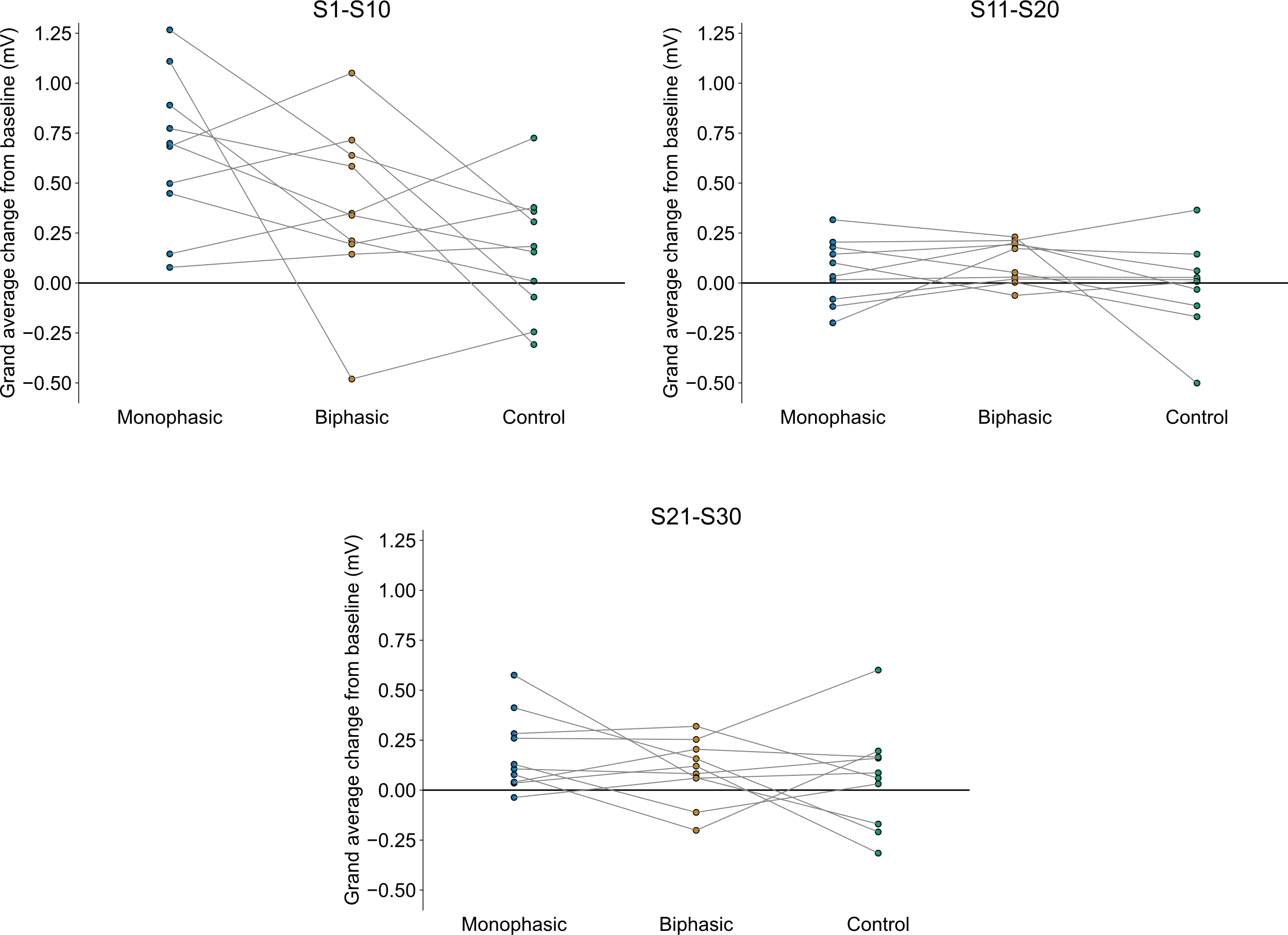
**

*Fig. S3: Group mean grand-average change in MEP amplitude compared to baseline across the 60-min post-iTBS time period for the M1 (monophasic and biphasic) and the control (vertex) condition. The sample (N=30) has been split randomly into thirds, to more clearly support visualization of each participant in the study and their trajectory across conditions.*


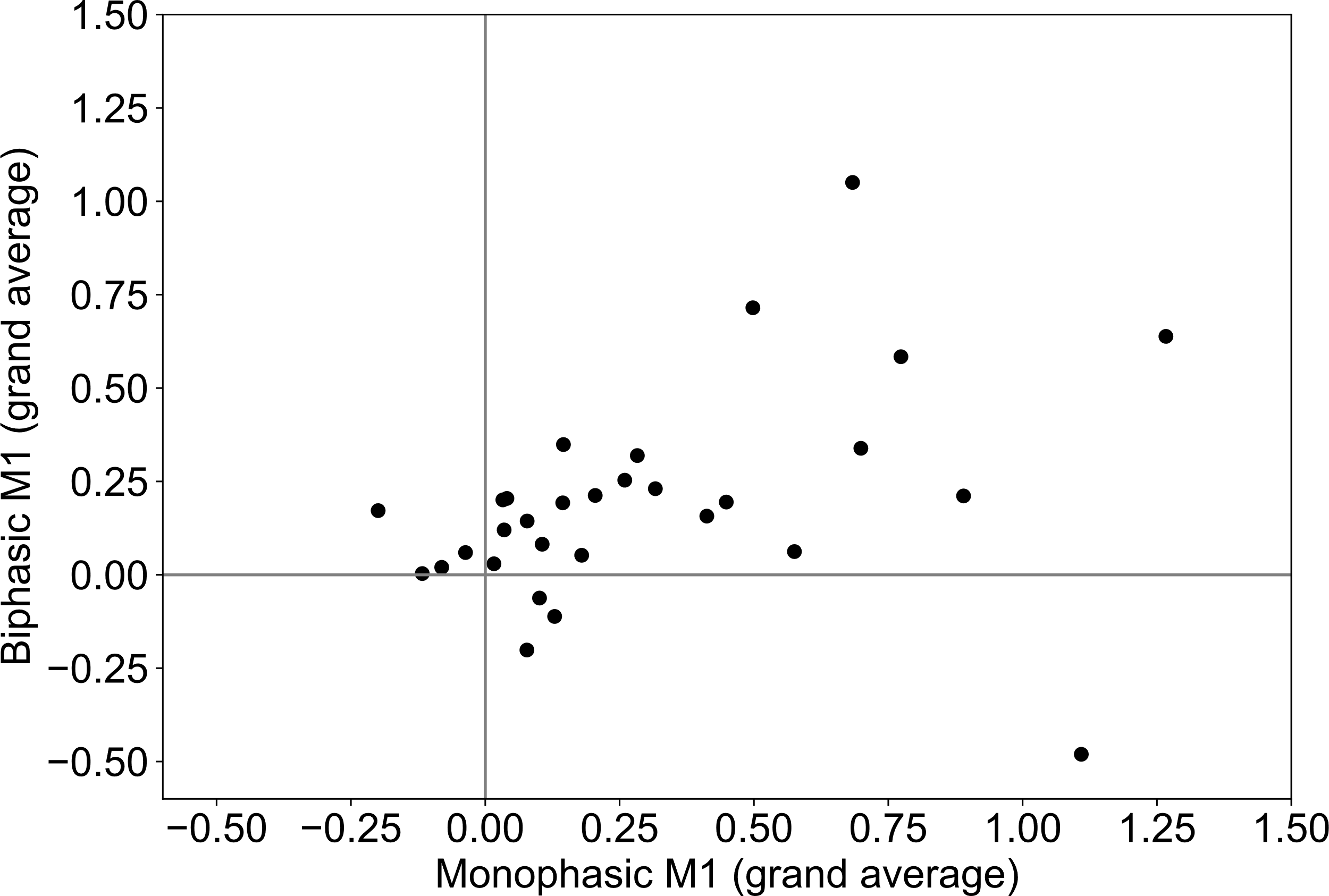


*Fig. S4: Group mean grand-average change in MEP amplitude compared to baseline across the 60-min post-iTBS time period for the biphasic M1 condition vs the monophasic M1 condition. Each participant is indicated by a dot. The non-responders in one condition are shown to not correspond to the non-responders in the other condition. For the majority of the data, participants respond in both conditions but overall, more strongly in the monophasic condition.*

*
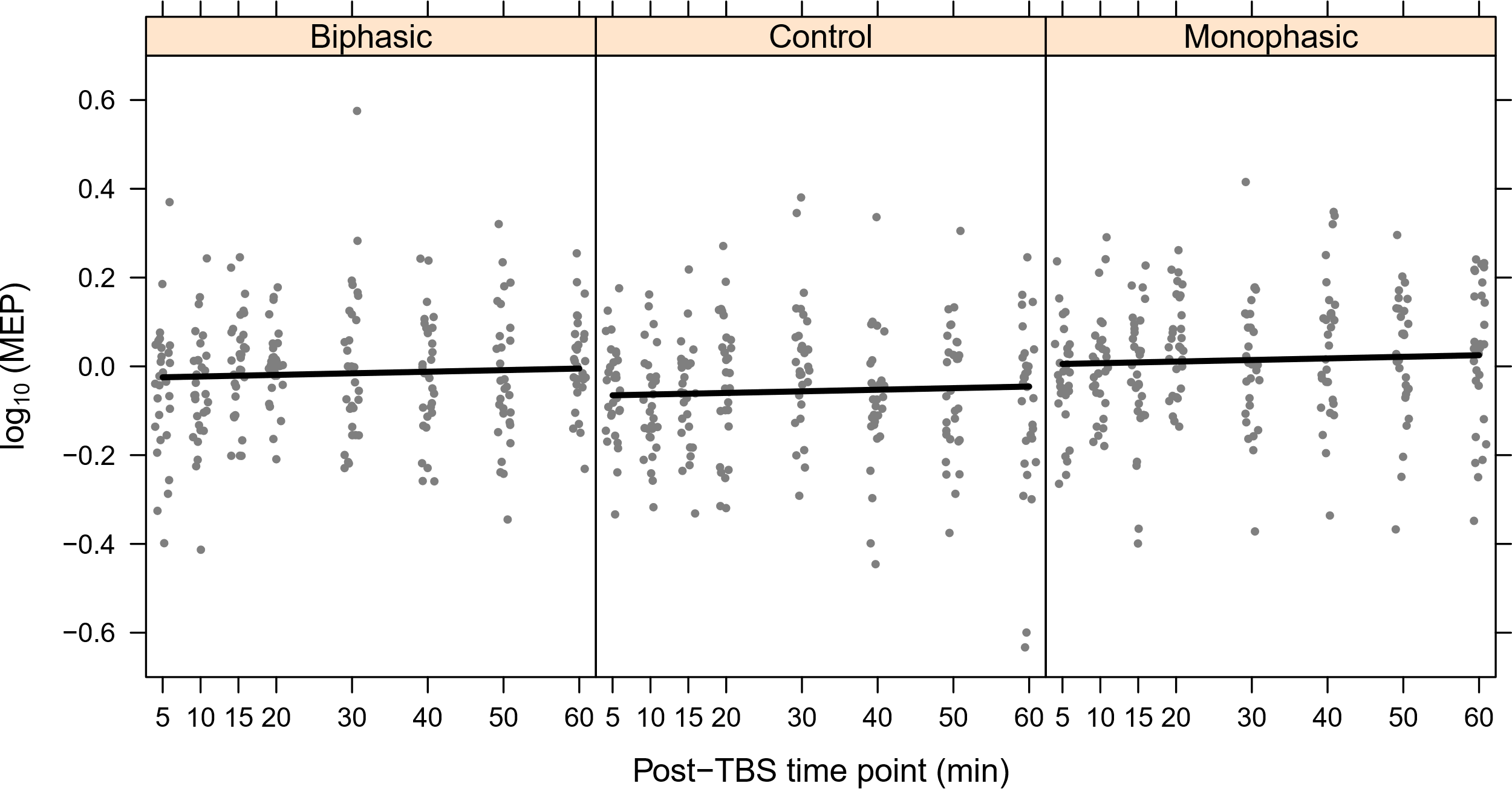
*

*Fig. S5: Visualisation of the fit of the linear mixed effect model (including the fixed effect of iTBS condition) to the data from the monophasic and biphasic M1 iTBS conditions and the vertex condition. The model was fit to* *the log-transformed absolute MEP amplitudes. The baseline data (not shown here) were modelled as a separate fixed effect. Solid lines show the model predictions, single dots show partial residuals as generated using the ‘visreg’ function in R.*

**Supplementary analysis using the participant block-wise mean of the MEP amplitudes instead of the median**

For each participant and each stimulation block the mean MEP amplitude was calculated. Individual trials with MEP amplitudes outside >2 SD of the mean MEP amplitude were removed. Test of normality revealed that across ~55% of blocks across participants, the data distributions departed significantly from normality (Shapiro-Wilk < 0.05). Log-transformation did not resolve this. In the primary analysis in the main manuscript block-wise medians and log transformation were used to resolve this problem, before proceeding to parametric analyses. However, here, for comparison, analyses on means are reported, per a reviewer’s request.

RmANOVA revealed a significant effect of Time (F(1, 29) = 28.478; p < 0.001) and Condition (F(2, 58) = 3.686; p = 0.041) but no interaction of Time and Condition (F(2, 58) = 1.914; p = 0.165). Holm-Bonferroni corrected pairwise comparisons showed that only the monophasic plasticity effect was significantly larger than the control condition (t(29) = 2.627; p = 0.041; dz = 0.293; monophasic vs biphasic: t(29) = 0.890; p = 0.381; dz = 0.088; biphasic vs control: t(29) = 1.626; p = 0.230; dz = 0.220).

To directly compare the effect of iTBS condition, the LME models with and without the fixed effect of iTBS condition were compared using likelihood ratio testing, which showed that the iTBS condition (monophasic M1, biphasic M1, vertex) had a significant effect on the MEP amplitude (χ2(1) = 31.948, p < 0.001). Post-hoc comparisons revealed significant differences between the monophasic and biphasic M1 conditions (t(693) = 2.377, p= 0.018), as well as the M1 conditions and the vertex condition (biphasic vs vertex: t(699) = 3.411, p = 0.001), monophasic vs vertex: t(702) = 5.738, p < 0.001).

**Supplementary analysis of the control condition**

In the control condition (iTBS applied to the vertex) participants were randomized 50:50 to receive either monophasic or biphasic pulses. Given the well-established spatial specificity of TMS, we predicted that iTBS applied several centimeters (~5-7cm) medial from M1 (i.e. over the vertex) would not induce a significant change in excitability of the motor corticospinal tract. Thus, we randomized participants to receive half monophasic and half biphasic iTBS to the vertex. Had we instead chosen all control pulses to be monophasic or biphasic only, one could wonder if the other untested control pulse shape might have induced different effects. To fully rule out potential bias from one or the other pulse shape in the control condition, we ran statistical analyses to test for any significant differences between these two halves of the control data set. Fig S6 shows the grand-average change from baseline, with the control condition data split by pulse type. An independent two-sample t-test confirmed there was no significant difference between the two vertex conditions (t(24.57) = 1.374, p = 0.182), reflected in the overlapping distributions of individual datapoints across the two pulse types.


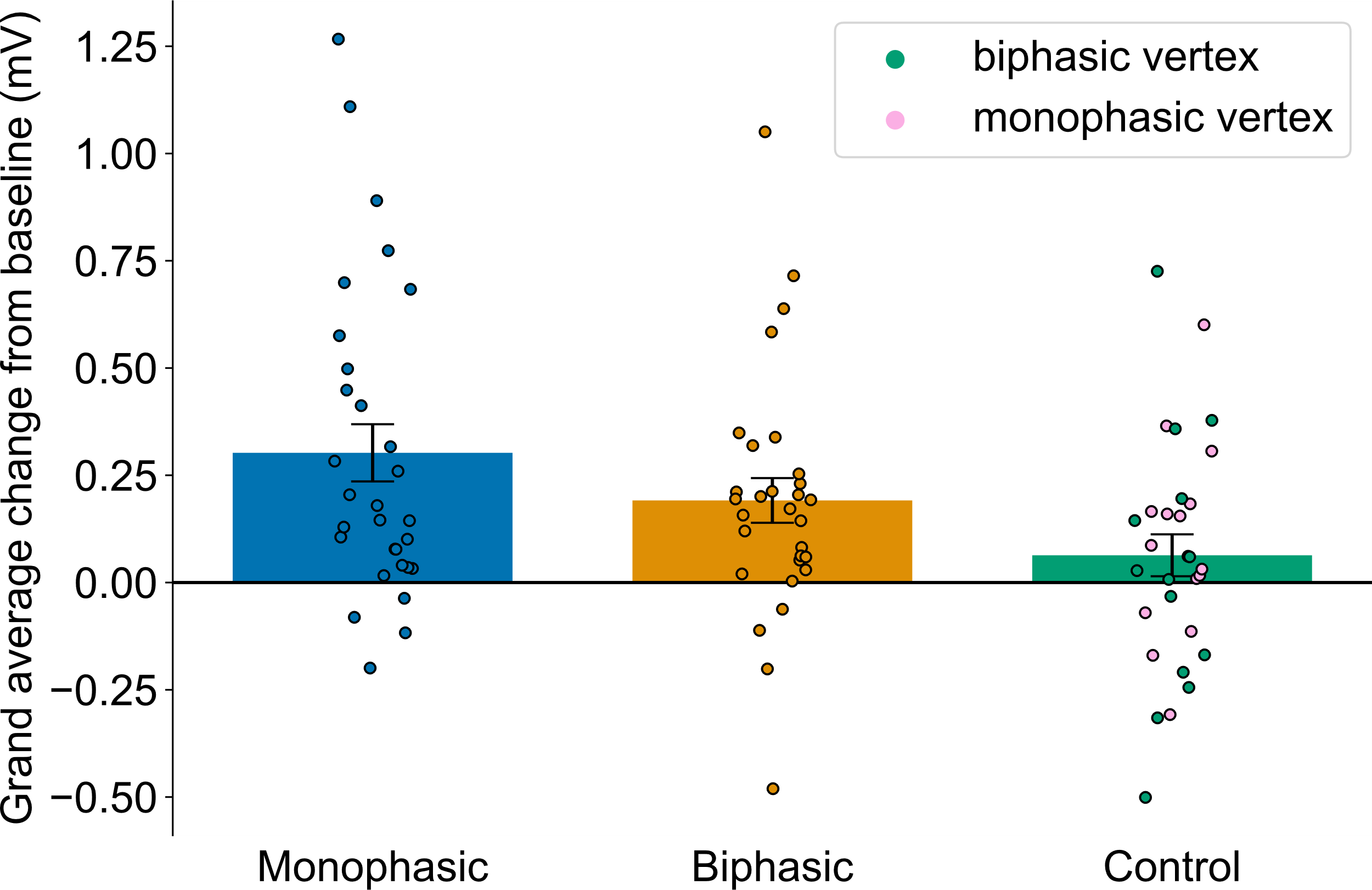


*Fig. S6: Group mean grand-average change in MEP amplitude compared to baseline across the 60-min post-iTBS time period for the M1 (monophasic and biphasic) condition and the control (vertex) condition. Individual participants are indicated by dots. Half of the participants received monophasic vertex stimulation (pink dots), and half biphasic (green). Note the overlapping distributions. The bars indicate group means and the error bars represent ± 1 standard error of the mean.*

In addition, we tested the full time-course data for any difference between the monophasic and biphasic vertex control datasets. Fig. S7 shows the data for each participant. Two LME models were built and contrasted, one included the fixed effect of pulse shape (mono- versus biphasic vertex iTBS) and the other did not. Likelihood ratio testing showed that the pulse shape in the control condition did not have a significant effect on the MEP amplitude (χ2(1) = 0.76, p = 0.383), further confirming no significant difference between the two halves of the vertex control dataset.

In combination, both analyses (Fig S6, S7) confirm there was no difference between the mono- and biphasic halves of the control dataset. Both were combined into a single vertex control condition for all analyses.


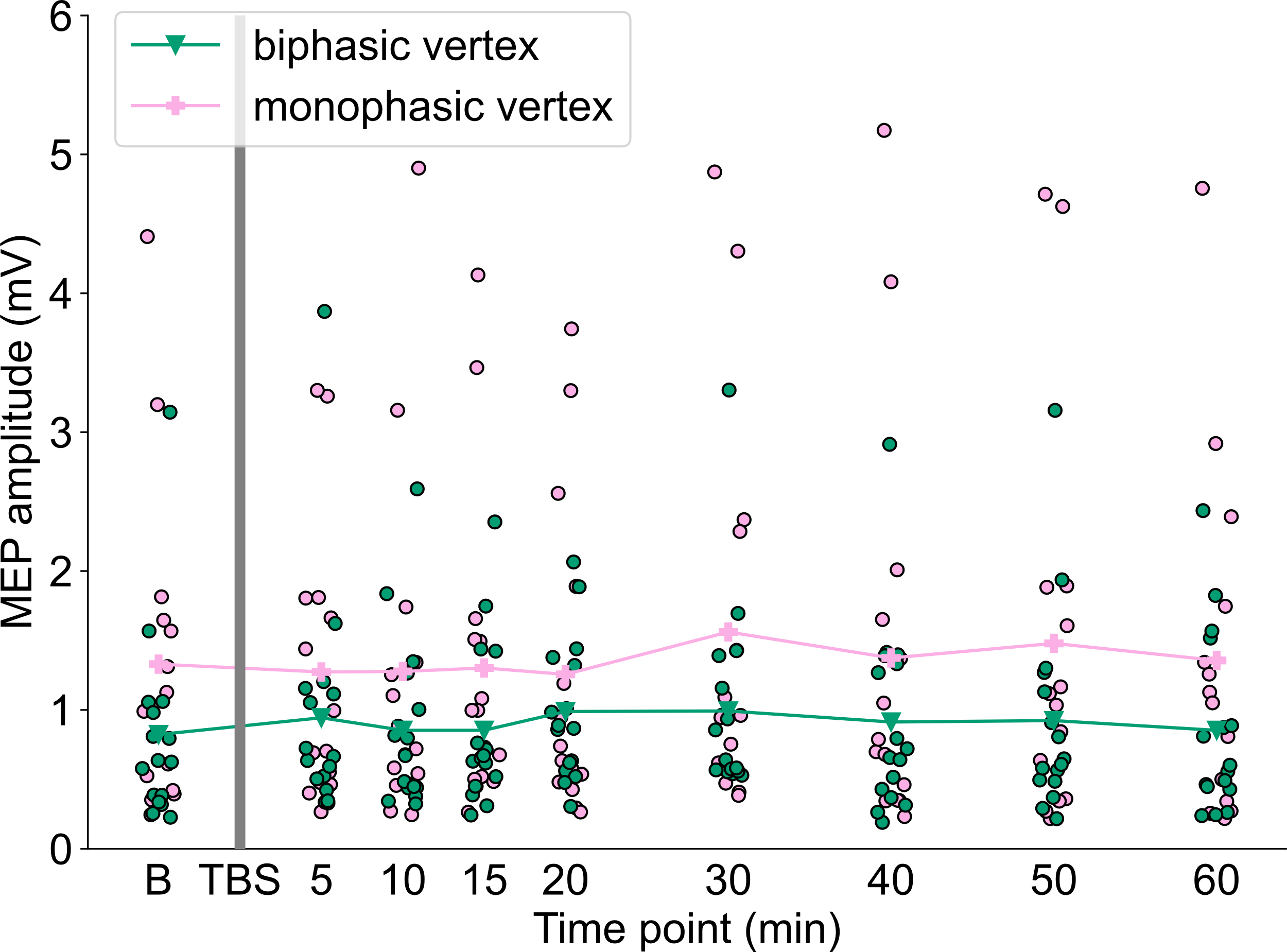


*Fig. S7: The group mean and individual participant MEP amplitudes over time for the control condition split for illustration into the two halves of the control dataset, i.e. those who had monophasic versus biphasic pulses in the control condition. The group means are indicated by the respective lines, the individual participants by dots. Monophasic data are in pink, biphasic in green. Note the overlapping data distributions, reflecting no significant difference between the two halves of the control condition.*
